# P01 The Australian experience of adapting and implementing ‘To Dip or Not to Dip’ in residential aged care facilities

**DOI:** 10.1093/jacamr/dlad077.004

**Published:** 2023-08-02

**Authors:** Lyn-li Lim, Melanie Wroth, Kathleen Williams, Juanita Breen

**Affiliations:** Aged Care Quality and Safety Commission, Victoria, Australia; Department of Infectious Diseases, University of Melbourne, Victoria, Australia; Aged Care Quality and Safety Commission, Victoria, Australia; Aged Care Quality and Safety Commission, Victoria, Australia; Aged Care Quality and Safety Commission, Victoria, Australia

## Abstract

**Objectives:**

‘To Dip or Not to Dip’ (TDONTD) is a behavioural intervention focused on reducing inappropriate antibiotic prescribing for urinary tract infections (UTI) by changing urine dipstick testing practice. It was adapted by the Aged Care Quality and Safety Commission from a successful initiative in England’s care homes. We describe TDONTD adaptation and evaluation of TDONTD to support improvement in UTI management in Australian residential aged care facilities.

**Methods:**

Data were collected in 12 facilities, from November 2021 to July 2022. Baseline and 3 month interviews were conducted with nurse and pharmacist champions implementing TDONTD and antibiotic prescribing audits.

**Results:**

Australia’s most widely accepted national guideline for assessment of residents with suspected UTI discourages urine testing in asymptomatic persons, however, is not explicit on when to (or not) perform dipstick testing in residents who do not meet minimum criteria for UTI. We adapted key resources including case-based education, clinical pathway for assessing residents for suspected UTI and antibiotic audit tool to incorporate national guideline recommendations around dipstick testing. Surveys with nurse champions of urine dipstick practice showed improved practice in the facilities related to reduced testing for asymptomatic bacteriuria (ASB) and increased use of formal protocol to guide dipstick testing practice and UTI diagnosis. Qualitative interviews with champions identified: (i) increased knowledge around ASB, clinical signs and symptoms of suspected UTI; (ii) increased confidence in not relying on dipstick testing to diagnose suspected UTI; (iii) TDONTD challenged and changed nurse perceptions of how residents present with UTI; (iv) pressure from external healthcare professional providers and family to perform dipstick testing was common; and (v) TDONTD was a feasible intervention in Australian RACF setting. Oral antibiotic prescribing for all indications, including UTI, improved with the intervention as did prescribing appropriateness for UTI treatment but not prophylaxis (Table 1).

**Table 1.** Oral antibiotic prescriptions for UTI at baseline, 3 months and 6 months

Oral antibiotic prescriptions	Baseline (%), 1074 residents	3 months (%), 1054 residents	6 months (%), 634 residents
For all indications	7.9	5.2	5.1
For UTI	3	2.6	1.6
By appropriateness (indication and duration)			
For UTI treatment	27	58	100
For UTI prophylaxis	6	18	14

**Conclusions:**

We found that TDONTD supported improved clinical management of residents with suspected UTI in an Australian aged care setting. Knowledge, skills and environmental context and resources contributed towards practice change. External healthcare professional providers and family were barriers to change suggesting that education around dipstick testing should be expanded to broader community and hospital healthcare settings in Australia.

